# Supplementary material for: Controlling light emission from semiconductor nanoplatelets using surface chemistry
Source: Nat Commun. 2024 Sep 4;15:7737. doi: 10.1038/s41467-024-51842-4 (PMC11374790; doi:10.1038/s41467-024-51842-4)
Supplement: Supplementary file 1 — Supplementary Information [file 41467_2024_51842_MOESM1_ESM.pdf]

Supplementary Information:
Controlling light emission from semiconductor nanoplatelets using surface chemistry

Michael W. Swift, Alexander L. Efros, and Steven C. Erwin

Center for Computational Materials Science,

Naval Research Laboratory, Washington, DC 20375, USA.

Suppl. Table 1. CdSe nanoplatelet parameters

Parameter	Value	Description
ϵ_∞	6.1	Interior high-frequency dielectric constant
ϵ_{o}	2.25	Exterior dielectric constant
m_{h}	0.190 m_0	Hole effective mass
a_{c}	0.608 nm	Cubic CdSe lattice constant
E_{p}	17.5 eV	Kane energy
E_{g}	1.66 eV	Bulk band gap
a_{x}	5.4 nm	Bulk exciton radius
C^{SR}	160 meV	Short-range exchange constant
$\hbar\omega_{\mathrm{LT}}^{\mathrm{bulk}}$	1.167 meV	Bulk longitudinal-transverse exciton splitting

Suppl. Table 2. Calculated thickness-dependent quantities for CdSe nanoplatelets

	2ML	3ML	4ML	5ML	6ML	7ML
d (nm)	0.608	0.912	1.22	1.52	1.82	2.13
M (m_0)	0.412	0.387	0.367	0.354	0.344	0.337
a_0 (nm)	1.37	1.62	1.85	2.05	2.24	2.42
$E_{\rm b}$ (eV)	−0.375	−0.299	−0.252	−0.220	−0.196	−0.178
$\phi_{\rm 2D}(0)^2$ (nm^{-2})	0.338	0.241	0.186	0.151	0.127	0.109
$\hbar\omega$ (eV)	3.17	2.69	2.42	2.25	2.12	2.03
$\hbar\omega_{\rm LT}$ (meV)	0.321	0.445	0.547	0.636	0.713	0.780
τ_0 (ns)	0.505	0.595	0.660	0.711	0.753	0.788
$\alpha^{\rm acetate-S}$ (eV)	0.528	0.316	0.237	0.215	0.159	0.144
$\alpha^{\rm acetate-Cl}$ (eV)	0.225	0.144	0.0915	0.0722	0.0454	0.0369
$\alpha^{\rm acetate-I}$ (eV)	0.352	0.204	0.117	0.0909	0.0560	0.0337
n_0 (meV^{-1})	0.775	0.727	0.691	0.666	0.647	0.633
W (meV)	11.1	3.73	1.99	1.58	0.843	0.672
R_W (nm)	2.88	5.14	7.22	8.25	11.5	13.0
L_W (nm)	12.0	16.6	18.7	19.4	20.8	21.2
$\varepsilon_{\rm m}$ (meV)	−5.81	−3.07	−2.00	−1.63	−0.956	−0.846
a_1	3.61	4.32	5.14	5.43	6.36	6.89
a_2	3.55	13.5	12.8	13.2	10.8	7.76
a_3	0.465	1.65	2.62	3.10	4.66	5.52
Δ (meV)	36.7	14.6	8.59	7.05	4.15	3.48
$\Delta E_{\rm exch}^{\rm SR}$ (meV)	30.0	14.3	8.26	5.37	3.75	2.77
$\Delta E_{\rm exch}^{\rm LR}$ (meV)	4.84	3.47	2.87	2.57	2.23	2.02
$\Delta E_{\rm exch}$ (meV)	34.8	17.7	11.1	7.94	5.98	4.78
$\mathcal{A}$	0.110	0.119	0.139	0.164	0.181	0.204
$\max(1/\tau^*(\varepsilon^*))$	1.05	0.638	0.476	0.424	0.318	0.287
$\tau_{\rm ex}^{\rm ideal}$ (ps)	2.53	4.17	5.99	7.94	10.0	12.2
$\min(\tau(\varepsilon))$ (ps)	17.6	16.0	16.5	19.4	17.4	18.8
$\min(\tau_{\rm avg}(T))$ (ns)	0.214	0.209	0.210	0.218	0.221	0.233
$T_{\tau\min}$ (K)	342	186	128	98	78	66

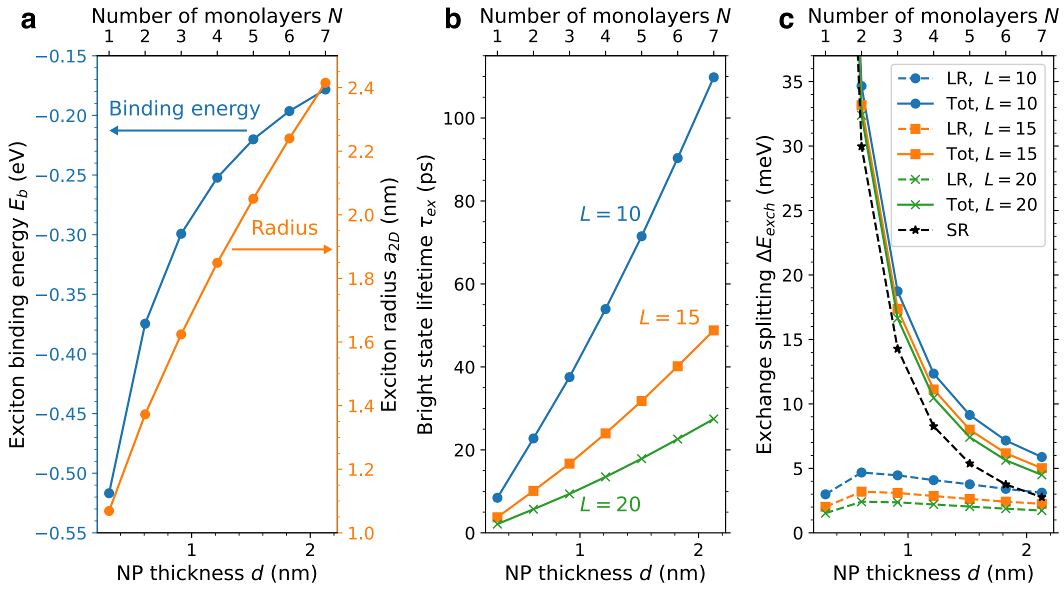

Suppl. Fig. 1. Optical characteristics of ideal CdSe nanoplatelets, in which exciton center-of-mass motion extends over the entire nanoplatelet. **a** The dependence of the 2D exciton binding energy E_b and radius a_2D on the nanoplatelet thickness d. **b** Thickness dependence of the bright exciton decay time τ_ex calculated for square nanoplatelets with side lengths L (in nm) as labeled on the figure. **c** Thickness dependence of the splitting between bright and dark excitons created by short-range (SR) and long-range (LR) exchange and their combined contribution as a function of nanoplatelet thickness. Short-range exchange splitting is independent of the lateral size of the nanoplatelet.

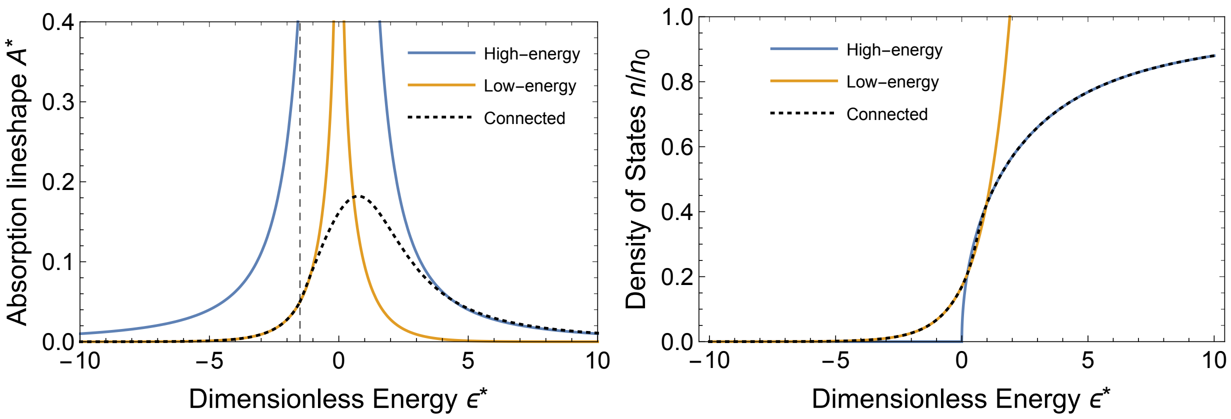

Suppl. Fig. 2. Connecting high-energy and low-energy limits. **a** The limiting behavior of $A^*(\varepsilon^*)$ at high energy ($A^* = 1/(\varepsilon^*)^2$, shown in blue) and low energy (eq. 18, shown in orange). The connected expression $A(\varepsilon)$ in eq. 9 is shown by a black dotted line. Similar matching occurs for numerical $A(\varepsilon)$ as discussed in Methods section 3 **b** The limiting behavior of $n(\varepsilon)$ at high energy (eq. 22, shown in blue) and low energy (eq. 21, shown in orange). The expression given in eq. 23 for $n(\varepsilon)$ is shown by a black dotted line.

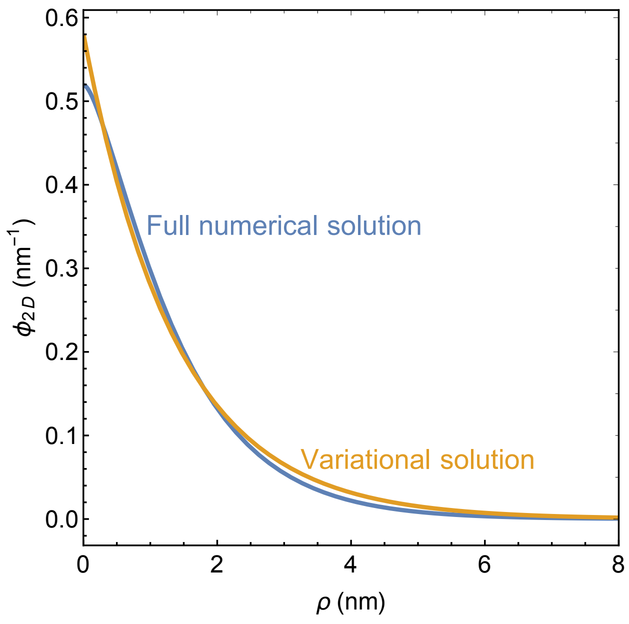

Suppl. Fig. 3. Comparison of the variational result for the internal wavefunction for 2 ML nanoplatelets, $\phi^d_{2\mathrm{D}}(\rho)$ (shown in orange) with the full numerical result (shown in blue). The energy of the variational solution is less than 2 meV higher than the energy of the full numerical solution.

Suppl. Note 1. Validating wavefunction of relative motion

To validate the variational ansatz, we also solved the Schrödinger equation numerically in the effective Hanamura potential, as described in ref. 12. The numerical binding energies were within 2 meV of the variational results, and as shown in Suppl. Fig. **3**, the wavefunctions are very similar. We choose to use the variational result for simplicity and transferability, since it is easily captured by the single parameter $a_{2\mathrm{D}}$.

The Hanamura potential expresses the effect of dielectric confinement as an infinite series of image charges resulting from the dielectric interface [39]. We used 16 images in our calculations, which we found to yield binding energies converged to within one meV and exciton radii converged to within 0.1% (Suppl. Fig. **4**). By contrast, the single-image method employed in ref. 7 overestimates the exciton radius by 7% for 7-monolayer platelets, and the error increases steadily for thinner nanoplatelets, reaching 25% for 1-monolayer platelets. This leads to an underestimation of the short-range exchange energy by 12% (0.23 meV) for 7-monolayer platelets, with error increasing steadily for thinner nanoplatelets, reaching 35% (23 meV) for 1-monolayer platelets.

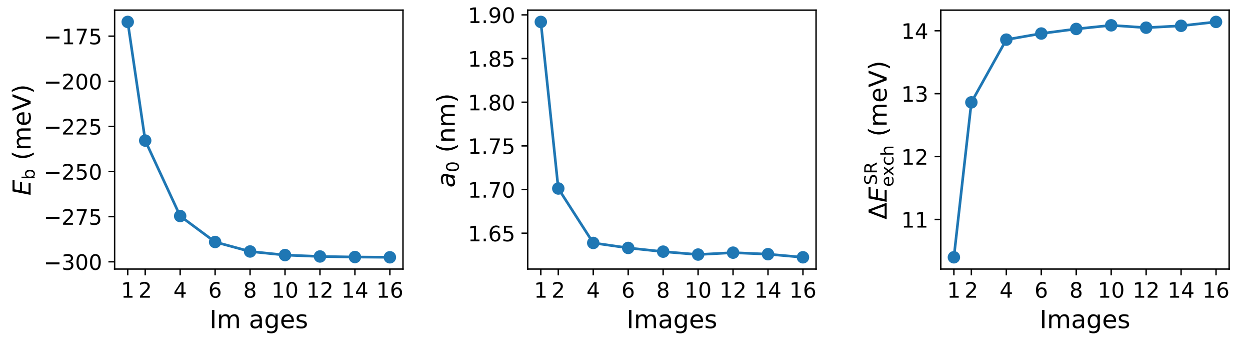

Suppl. Fig. 4. Convergence of the binding energy, E_b, the exciton radius $a_{2\mathrm{D}}$, and the short-range fine-structure splitting $\Delta E^{\mathrm{SR}}_{\mathrm{exch}}$. Using 16 images, the exciton binding energy is converged to less than 1 meV and the radius is converged to within approximately 0.1%, within the noise of our numerical integration.

Suppl. Note 2. Fine structure

The form of the exciton exchange interactions depends on the band-edge Bloch functions. For the conduction band in CdSe, they are [40]

$$u^{\mathrm{c}}_{1/2,1/2} = S\uparrow, \qquad u^{\mathrm{c}}_{1/2,-1/2} = S\downarrow . \tag{S1}$$

The Bloch functions of the valence band are

$$\begin{aligned} u^{\mathrm{v}}_{3/2,3/2} &= -\frac{1}{\sqrt{2}}(X+iY)\uparrow, & u^{\mathrm{v}}_{3/2,-3/2} &= \frac{1}{\sqrt{2}}(X-iY)\downarrow \\ u^{\mathrm{v}}_{3/2,1/2} &= \frac{1}{\sqrt{6}}[-(X+iY)\downarrow +2Z\uparrow], & u^{\mathrm{v}}_{3/2,-1/2} &= \frac{1}{\sqrt{6}}[(X-iY)\uparrow +2Z\downarrow]. \end{aligned} \tag{S2}$$

Confinement in a nanoplatelet increases the energy of the Z-containing light-hole states $u^{\mathrm{v}}_{3/2,\pm1/2}$, so we assume the ground state will be made up of heavy-hole $u^{\mathrm{v}}_{3/2,\pm3/2}$ states.

2 A. Short-range exchange

The short-range exchange Hamiltonian, neglecting constant terms, is [47]

$$\hat{H}^{\mathrm{SR}} = -\frac{2}{3}C^{\mathrm{SR}}\Omega(\boldsymbol{\sigma}_\mathrm{e}\cdot\boldsymbol{J})\delta(\boldsymbol{r}_\mathrm{e}-\boldsymbol{r}_\mathrm{h}) \ , \tag{S3}$$

where $\boldsymbol{\sigma}_\mathrm{e}$ is the electron spin-1/2 Pauli matrix and $\boldsymbol{J}$ is the hole angular momentum matrix. In the basis $u^{\mathrm{c}}_{1/2,1/2}u^{\mathrm{v}}_{3/2,3/2}$, $u^{\mathrm{c}}_{1/2,1/2}u^{\mathrm{v}}_{3/2,-3/2}$, $u^{\mathrm{c}}_{1/2,-1/2}u^{\mathrm{v}}_{3/2,3/2}$, $u^{\mathrm{c}}_{1/2,-1/2}u^{\mathrm{v}}_{3/2,-3/2}$, which we can

abbreviate $\uparrow\uparrow, \uparrow\downarrow, \downarrow\uparrow, \downarrow\downarrow$, the matrix representation of the Hamiltonian can be written

$$\tilde{H}^{\rm SR} = -\frac{2}{3}C^{\rm SR}\Omega \begin{pmatrix} \frac{3}{4} & 0 & 0 & 0 \\ 0 & -\frac{3}{4} & 0 & 0 \\ 0 & 0 & -\frac{3}{4} & 0 \\ 0 & 0 & 0 & \frac{3}{4} \end{pmatrix} \delta(\boldsymbol{r}_{\rm e}-\boldsymbol{r}_{\rm h}) = C^{\rm SR}\Omega \left(-\frac{1}{2}\mathbb{I} + \begin{pmatrix} 0 & 0 & 0 & 0 \\ 0 & 1 & 0 & 0 \\ 0 & 0 & 1 & 0 \\ 0 & 0 & 0 & 0 \end{pmatrix} \right) \delta(\boldsymbol{r}_{\rm e}-\boldsymbol{r}_{\rm h}) \tag{S4}$$

The wavefunction envelope of an exciton confined to a square nanoplatelet of thickness d, and side-length L is

$$\Psi_d(\boldsymbol{r}_{\rm e},\boldsymbol{r}_{\rm h}) = \frac{4}{dL}\cos\left(\frac{\pi X}{L}\right)\cos\left(\frac{\pi Y}{L}\right)\cos\left(\frac{\pi z_{\rm e}}{d}\right)\cos\left(\frac{\pi z_{\rm h}}{d}\right)\phi^d_{\rm 2D}(\boldsymbol{r}_{\rm e}-\boldsymbol{r}_{\rm h}) \ , \tag{S5}$$

where X and Y are the center-of-mass coordinates of the exciton. We define the overlap integral Θ by integrating over the nanoplatelet [14]

$$\Theta = \Omega \iint_V d^3r_{\rm e}d^3r_{\rm h}\Psi^*_d(\boldsymbol{r}_{\rm e},\boldsymbol{r}_{\rm h})\delta(\boldsymbol{r}_{\rm e}-\boldsymbol{r}_{\rm h})\Psi_d(\boldsymbol{r}_{\rm e},\boldsymbol{r}_{\rm h}) = \frac{3}{2d}\Omega|\phi^d_{\rm 2D}(0)|^2 \ . \tag{S6}$$

Therefore, the splitting between the lowest exciton levels due to short-range exchange is

$$\Delta E^{\rm SR}_{\rm exch} = C^{\rm SR}\Theta = C^{SR}\Omega\frac{3}{2d}|\phi^d_{\rm 2D}(0)|^2 \tag{S7}$$

Note that the factor of 3/2 from the overlap integral is missing from the corresponding equation 6 in ref. 7. The short-range exchange constant for cubic CdSe is $C^{\rm SR} = 160$ meV [7]. The unit cell volume is $\Omega = a_{\rm c}^3 = 0.225$ nm^3.

2 B. Long-range exchange

The long-range exchange interaction for a state X_i in a nanoplatelet is described by the Hamiltonian [14, 48]

$$H^{LR}_{X_i} = |\tilde{\boldsymbol{p}}_{X_i}|^2\frac{1}{\epsilon_\infty}\left(\frac{P_{\rm cv}\hbar e}{m_0\hbar\omega}\right)^2\frac{4\pi}{3}\frac{\Theta}{\Omega}\mathcal{A}_{X_i} \ , \tag{S8}$$

where $\tilde{\boldsymbol{p}}_{X_i}$ is the dimensionless unit cell momentum matrix element, ϵ_∞ is the high-frequency dielectric constant, $P_{\rm cv}$ is the Kane matrix element (related to the Kane energy through $E_{\rm p} = 2|P_{\rm cv}|^2/m_0$), and $\hbar\omega$ is the optical transition energy. The overlap integral Θ is defined by eq. S6. For CdSe, the bulk and nanoplatelet Bloch functions both have the same form given by eqs. S1,S2, which gives $|\tilde{\boldsymbol{p}}_{X_i}| = 1$. We now define the envelope function

$$f(X,Y,z) = \frac{4}{dL}\phi^d_{\rm 2D}(0)\cos\left(\frac{\pi X}{L}\right)\cos\left(\frac{\pi Y}{L}\right)\cos^2\left(\frac{\pi z}{d}\right) \ . \tag{S9}$$

The dimensionless anisotropy function $\mathcal{A}_X(d/L)$ was calculated for in-plane polarized states in square nanoplatelets in ref. 14, and depends only on the ratio between the nanoplatelet thickness d and side length L:

$$\mathcal{A}_X = \frac{3}{4\pi}\frac{\Omega}{\Theta}\int_{V_1} dV_1 \int_{V_2} dV_2 \, [-\boldsymbol{\nabla}_1 \cdot f(\boldsymbol{r}_1)\hat{\boldsymbol{n}}_{X_i}]^* \, \frac{1}{|\boldsymbol{r}_1 - \boldsymbol{r}_2|} \, [-\boldsymbol{\nabla}_2 \cdot f(\boldsymbol{r}_2)\hat{\boldsymbol{n}}_{X_i}] \tag{S10}$$

$$= \frac{d}{2\pi}|\phi^d_{2\mathrm{D}}(0)|^{-2}\int_{V_1} dV_1 \int_{V_2} dV_2 \, [-\boldsymbol{\nabla}_1 \cdot f(\boldsymbol{r}_1)\hat{\boldsymbol{n}}_{X_i}]^* \, \frac{1}{|\boldsymbol{r}_1 - \boldsymbol{r}_2|} \, [-\boldsymbol{\nabla}_2 \cdot f(\boldsymbol{r}_2)\hat{\boldsymbol{n}}_{X_i}] \ . \tag{S11}$$

The LR exchange can be recast in terms of the bulk LT splitting in CdSe, which is [40]

$$\hbar\omega_{\mathrm{LT}}^{\mathrm{bulk}} = \frac{4}{\epsilon_\infty a_{\mathrm{x}}^3}\left(\frac{P_{\mathrm{cv}}\hbar e}{m_0 E_{\mathrm{g}}}\right)^2 \ , \tag{S12}$$

where a_{x} is the bulk exciton radius. We will use the experimental value for the bulk LT splitting. In wurtzite CdSe, it has been measured to be 0.95 meV. The band gap of wurtzite CdSe is 1.84 eV, compared to 1.66 eV for zincblende. Since the LT splitting is inversely proportional to the square of the band gap, we estimate the bulk LT splitting in zincblende CdSe to be 1.167 meV.

Substituting into eq. S8, the long-range exchange energy of the bright state is

$$\Delta E_{\mathrm{exch}}^{\mathrm{LR}} = \hbar\omega_{\mathrm{LT}}^{\mathrm{bulk}} a_{\mathrm{x}}^3 \left(\frac{E_{\mathrm{g}}}{\hbar\omega}\right)^2 \frac{\pi}{3}\frac{\Theta}{\Omega}\mathcal{A}(d) \tag{S13}$$

$$\Delta E_{\mathrm{exch}}^{\mathrm{LR}} = \hbar\omega_{\mathrm{LT}}^{\mathrm{bulk}} \left(\frac{E_{\mathrm{g}}}{\hbar\omega}\right)^2 \frac{\pi a_{\mathrm{x}}^3}{2d}|\phi^d_{2\mathrm{D}}(0)|^2\mathcal{A}(d) \tag{S14}$$

We note that eq. S8 is the same in CdSe as in the perovskites. However, when expressed in terms of the bulk LT splitting, the LR exchange splitting in perovskites appears larger by a factor of 3/2. This is because the bulk LT splitting in perovskites is reduced by a factor of $|\tilde{\boldsymbol{p}}_{X_i}|^2 = 2/3$.

An important piece of the LR exchange is the photon energy $\hbar\omega$. We use a physically-motivated empirical fit [14]:

$$\hbar\omega = A + C/d^2 + \left[B/(D + 1/d^2)\right]/d \ . \tag{S15}$$

With $\hbar\omega$ expressed in meV and d expressed in nm, a fit to the photoluminescence data [2, 13] yields $A = 1.443$, $B = 3.207$, $C = 0.272$, and $D = 2.633$. See Suppl. Fig. **5**.

In order to calculate $\mathcal{A}(d)$, we must describe the center-of-mass confinement of the exciton. In the absence of disorder, the exciton moves coherently over the whole nanoplatelet. This is

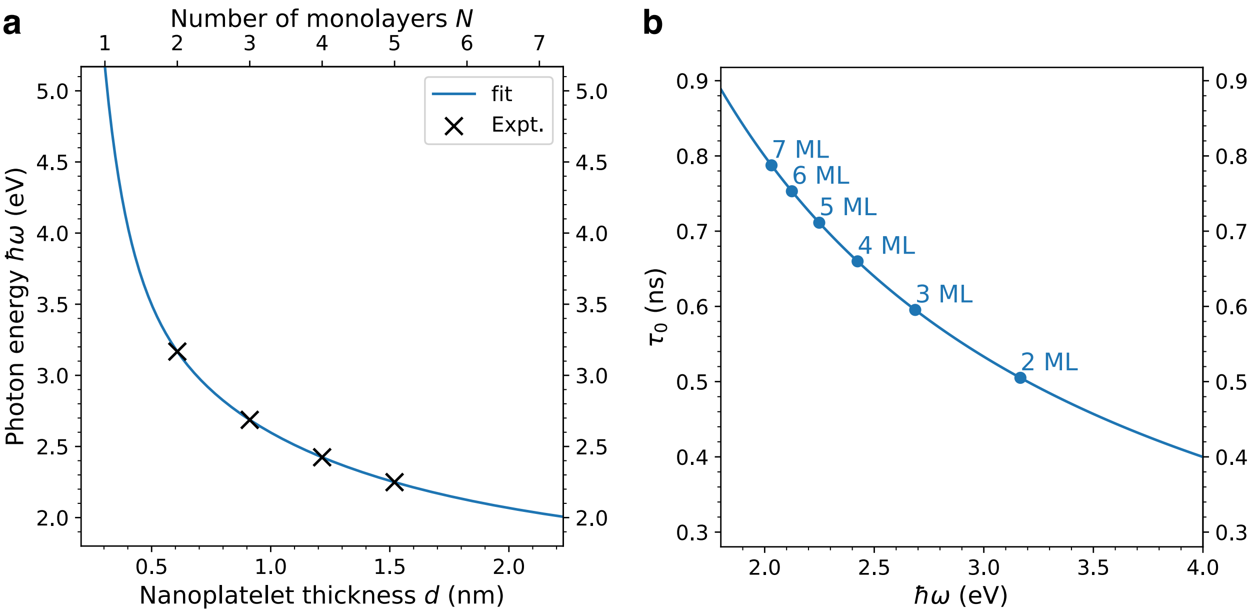

Suppl. Fig. 5. **a** Photon energy described by eq. S15, fitted to experimental photoluminescence data [2, 13]. **b** Characteristic lifetime τ_0 as a function of photon energy, as described by eq. 32.

the case illustrated in Suppl. Fig. **1c**. There, $\mathcal{A}(d)$ is based on square confinement using the model from Ref [14], with varying nanoplatelet side-lengths L given in the figure legend. In the presence of disorder, the exciton is localized by the random potential. The wavefunction of localization for energy ε is given by eq. 24, reproduced here:

$$\psi(\boldsymbol{R}, \boldsymbol{R}_f, R_\varepsilon) = \mathcal{N}(\boldsymbol{R}_f, R_\varepsilon)\, e^{-\left|\boldsymbol{R}-\boldsymbol{R}_f\right|/R_\varepsilon} \cos\left(\frac{\pi X}{L_x}\right) \cos\left(\frac{\pi Y}{L_y}\right) \tag{S16}$$

As before $R_\varepsilon = \hbar/\sqrt{2M\varepsilon}$ (eq. 13). The characteristic length scale is

$$L_\varepsilon = \left|\int d^2\boldsymbol{R}\; \psi_\varepsilon(\boldsymbol{R})\right| \tag{S17}$$

The energy of the typical localization is W, so the typical length scale of localization is L_W, plotted in Suppl. Fig. **6b**. We approximate $\mathcal{A}$ for long-range exchange using the square nanoplatelet model from ref. 14 with side length L_W.

2 C. Anisotropic LR exchange

CdSe nanoplatelets tend to be anisotropic [7]. In the absence of disorder, the exciton center-of-mass wavefunction will extend over the whole nanoplatelet. The exciton envelope

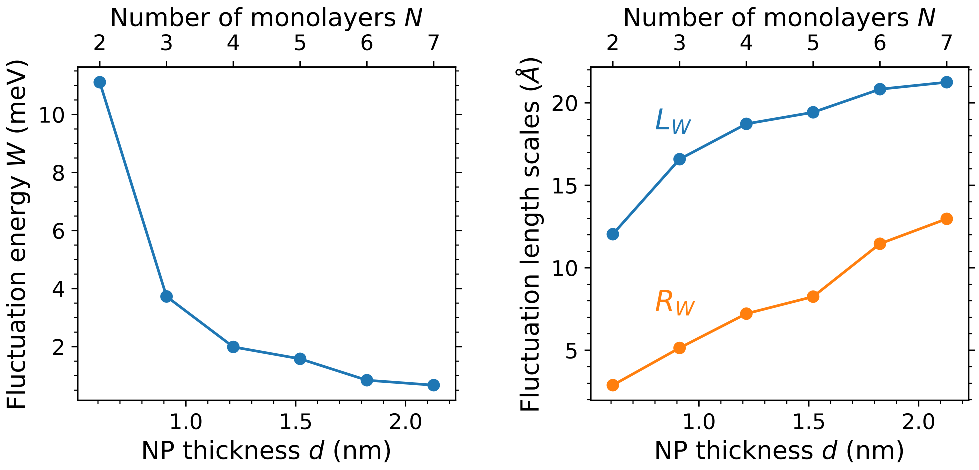

Suppl. Fig. 6. **a** Energy scale of potential fluctuations W (eq. 11) as a function of nanoplatelet thickness; **b** exciton center-of-mass localization radius L_W (eq. S17) and exponential decay length R_W (eq. 13) due to the random potential.

function is [14]

$$\Psi^d_{j_e,j_h}(\boldsymbol{r}_e,\boldsymbol{r}_h)=\frac{4u_{j_e}(\boldsymbol{r}_e)u_{j_h}(\boldsymbol{r}_h)}{d\sqrt{L_xL_y}}\cos\left(\frac{\pi X}{L_x}\right)\cos\left(\frac{\pi Y}{L_y}\right)\cos\left(\frac{\pi z_e}{d}\right)\cos\left(\frac{\pi z_h}{d}\right)\phi^d_{2D}(\boldsymbol{r}_e-\boldsymbol{r}_h)\ . \tag{S18}$$

Now defining

$$f(X,Y,z)=\frac{4}{d\sqrt{L_xL_y}}\phi^d_{2D}(0)\cos\left(\frac{\pi X}{L_x}\right)\cos\left(\frac{\pi Y}{L_y}\right)\cos^2\left(\frac{\pi z}{d}\right)\ , \tag{S19}$$

The LR exchange splitting in an anisotropic nanoplatelet is proportional to

$$\Delta E^{\mathrm{LR}}_{X_i}\propto\int_{V_1}dV_1\int_{V_2}dV_2\frac{[df(X_1,Y_1,z_1)/dX_{i,1}]^*\,[df(X_2,Y_2,z_2)/dX_{i,2}]}{\sqrt{(X_1-X_2)^2+(Y_1-Y_2)^2+(z_1-z_2)^2}}\ . \tag{S20}$$

We see that the long-range exchange interaction will split the two-fold degenerate bright state into states with transition dipoles aligned on the two axes of the nanoplatelet. Defining dimensionless coordinates $X'=X/L_x$, $Y'=Y/L_y$, and $z'=z/d$, and defining the ratios $r_x=d/L_x, r_y=d/L_y$, we make the appropriate modifications of ref. 14 to arrive at the following form of $\mathcal{A}_X$ and $\mathcal{A}_Y$:

$$\frac{\mathcal{A}_X}{8\pi r_x^2}=\frac{\mathcal{A}_Y}{8\pi r_y^2}=\int_{-1/2}^{1/2}dX'_1dY'_1dz'_1dX'_2dY'_2dz'_2 \tag{S21}$$

$$\frac{\sin(\pi X'_1)\cos(\pi Y'_1)\cos^2(\pi z'_1)\sin(\pi X'_2)\cos(\pi Y'_2)\cos^2(\pi z'_2)}{\sqrt{r_y^2(X'_1-X'_2)^2+r_x^2(Y'_1-Y'_2)^2+r_x^2r_y^2(z'_1-z'_2)^2}}\ .$$

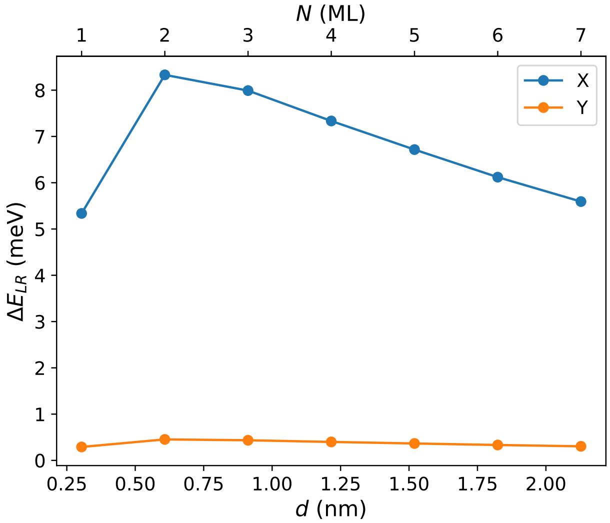

Suppl. Fig. 7. Long-range exchange interaction in an anisotropic nanoplatelet with $L_x = 7$ nm and $L_y = 30$ nm in the absence of disorder. The bright state is split into X-polarized (in blue) and Y-polarized (in orange).

Therefore, the ratio of LR exchange splitting for the states with transition dipoles polarized along the two directions is proportional to the inverse square of the side-length ratio:

$$\frac{\Delta E_X^{\mathrm{LR}}}{\Delta E_Y^{\mathrm{LR}}} = \left(\frac{L_Y}{L_X}\right)^2 \tag{S22}$$

As an example, Suppl. Fig. **7** shows the LR exchange in an anisotropic rectangular nanoplatelet with $L_x = 7$ nm and $L_y = 30$ nm in the absence of disorder-induced localization.

2 D. Comparison with previous fine-structure reports

Our calculated exchange splitting is significantly larger than earlier theoretical results [7]. The prior underestimation of the splitting was due to neglect of long-range exchange, an incorrect prefactor for short-range exchange (Suppl. Note 2A), and overestimation of the exciton radius arising from truncation of the dielectric confinement image series (Suppl. Note 1). Suppl. Fig. **8a** shows that the activation energies measured in ref. 7 lie between the transverse and longitudinal branches of the acoustic phonons (the confined phonon energy is estimated as $E_{\mathrm{ph}} = v\pi\hbar/d$, where the speeds of transverse and longitudinal phonons in CdSe are $v_{\mathrm{t}} = 1.54 * 10^5$ cm/s and $v_{\mathrm{l}} = 3.7 * 10^5$ cm/s respectively [54]). These measurements

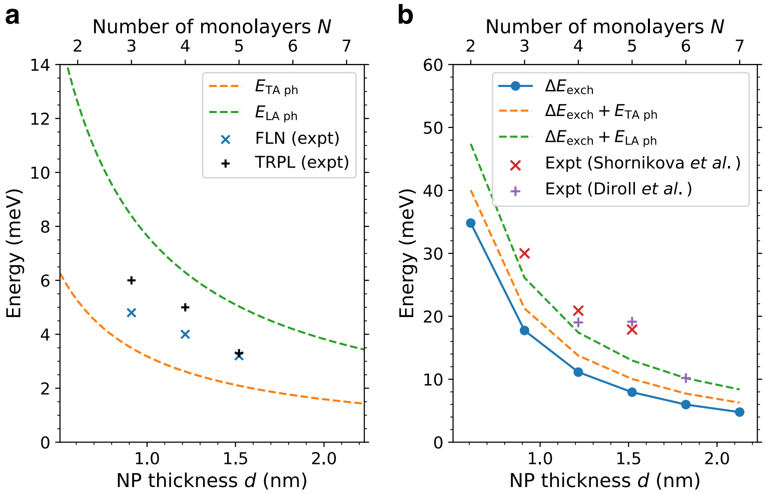

Suppl. Fig. 8. **a** Comparison of theoretical acoustic phonon energies with experimental activation energies that were previously interpreted as dark/bright exciton level separation[7, 10]. The experimental data are from ref. 7, and were extracted from fluorescence line narrowing (FLN, blue x's) and temperature-dependent time-resolved photoluminescence (TRPL, black +'s). TA phonon energies are shown as orange and LA as green curves. **b** Comparison of the experimental peak separation to the exciton fine structure calculated theoretically. Experimental results are from Refs. 7 (red x) and 52 (purple +). The sum of short-range exchange and long-range exchange is shown by the blue solid line. Dashed lines show the exchange energy plus acoustic-phonon energies: TA in orange and LA in green as in panel **a**. The remarkable agreement suggests that the lower-lying peak is due to LA-phonon-assisted recombination of the dark exciton state.

may not have detected the exchange splitting, but may instead have measured the activation of photoluminescence as dark excitons transfer to the low-energy tail of the bright exciton states *via* LA and TA phonons.

Other experimental evidence may also support our calculated exchange splitting. Dark excitons give rise to emission lines in CdSe quantum dots [46] and in halide perovskite nanoplatelets [14], so a dark exciton peak is also possible in CdSe nanoplatelets. Such a peak would lie below the bright exciton peak, with an energy separation equal to $\Delta E_{\mathrm{exch}} + E_{\mathrm{ph}}$, where E_{ph} is the energy of an emitted phonon, as shown in Suppl. Fig. **8b**. We expect the LA phonon branch to be the most relevant because its coupling to the radiative recombination of the dark state is much stronger than that of the TA phonon [55].

Remarkably, an emission peak fitting this description has already been observed. While

the emission spectrum of CdSe nanoplatelets shows a single narrow peak at room temperature [2, 35], two distinct emission peaks are observed below 160K, with an energy separation ranging from 18 meV for 5 ML nanoplatelets to 30 meV for 3 ML nanoplatelets. [7, 26, 50, 51] The high-energy line arises from neutral exciton recombination,[7, 50] but the origin of the low-energy line is still actively debated. Among the alternatives considered are LO-phonon-assisted recombination of an exciton, [26] trion emission,[7, 10, 12, 51] emission from excimer states in neighboring nanoplatelets,[52] and recombination from a ground p-type exciton state. [53] Our results suggest the low-energy peak may be due to phonon-assisted recombination of the dark exciton. A possible dark-exciton origin of the low-energy line is consistent with its slow decay at 4.2 K. [10]. The experimental peak splitting from Refs 7 and 10 is overlaid with our theoretical prediction in Suppl. Fig. **8b**. The agreement is striking. While this is far from conclusive evidence, the possibility merits further investigation.

Suppl. Note 3. DFT Details

Suppl. Fig. **9** shows atomistic visualizations of the 4 ML nanoplatelets; nanoplatelets with other thicknesses are similar.

In the acetate-, chlorine-, and iodine-passivated slabs, the highest-occupied and lowest-unoccupied states corresponded to the CdSe VBM and CBM, respectively. For the sulfur-passivated slabs, surface states were also present. The eigenvalues corresponding to the confined bulk-like CdSe band-edge states were identified manually based on the orbital projections, as shown in Suppl. Fig. **10**. Cd s projections were used to identify CBM states, and Se p projections were used to identify VBM states. The bulk-like states show a shape characteristic of the z-confinement envelope function. The energy difference between the identified CBM and VBM eigenvalues was identified as the gap.

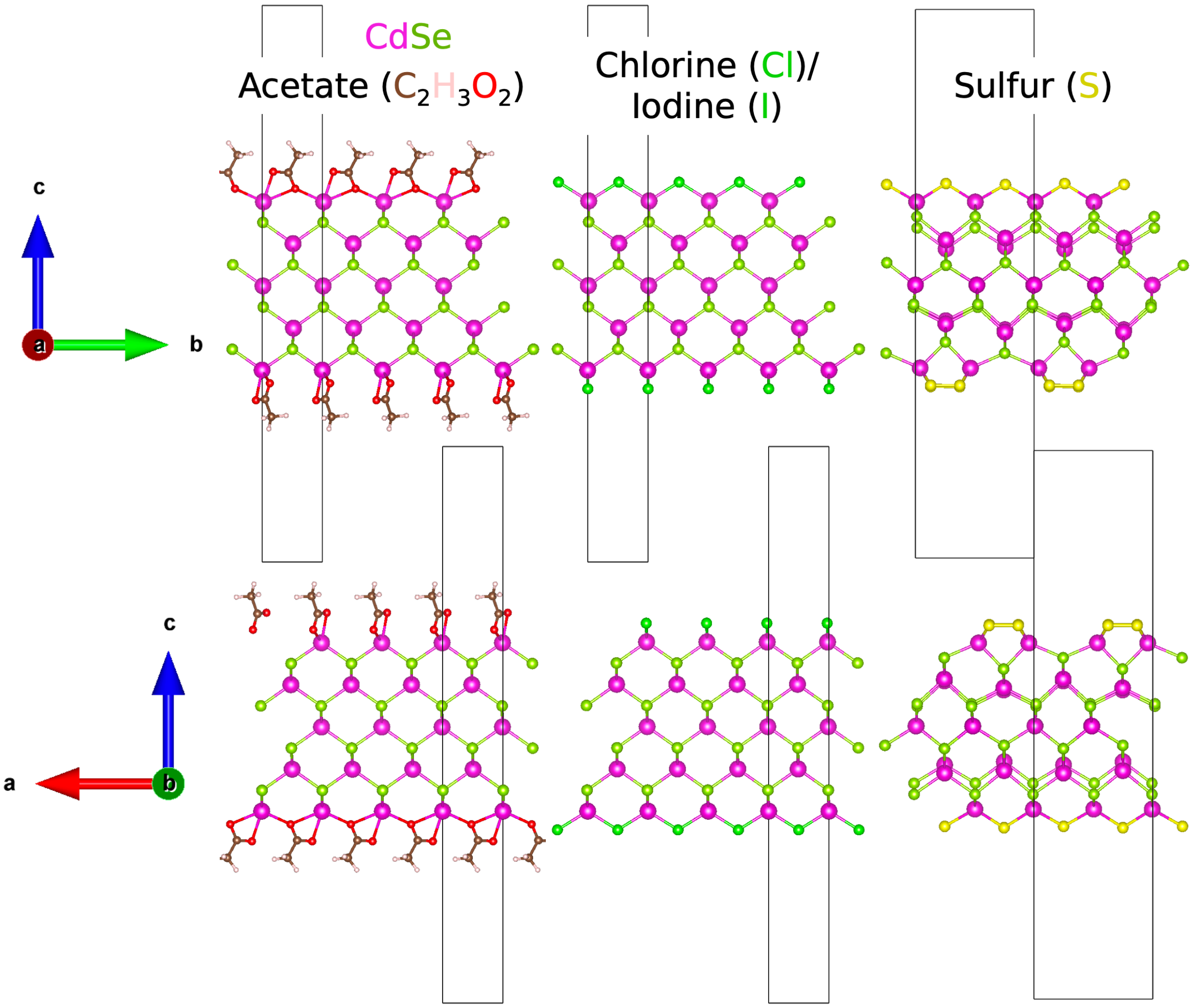

Suppl. Fig. 9. Atomic visualizations of DFT-calculated 4 ML nanoplatelets. The z direction pointing upward. The periodic unit cells are shown as black boxes. The top row shows the perspective looking down the a axis, while the bottom row looks down the b axis. The columns show differing passivation: acetate, chlorine (iodine is visually identical), and sulfur passivation.

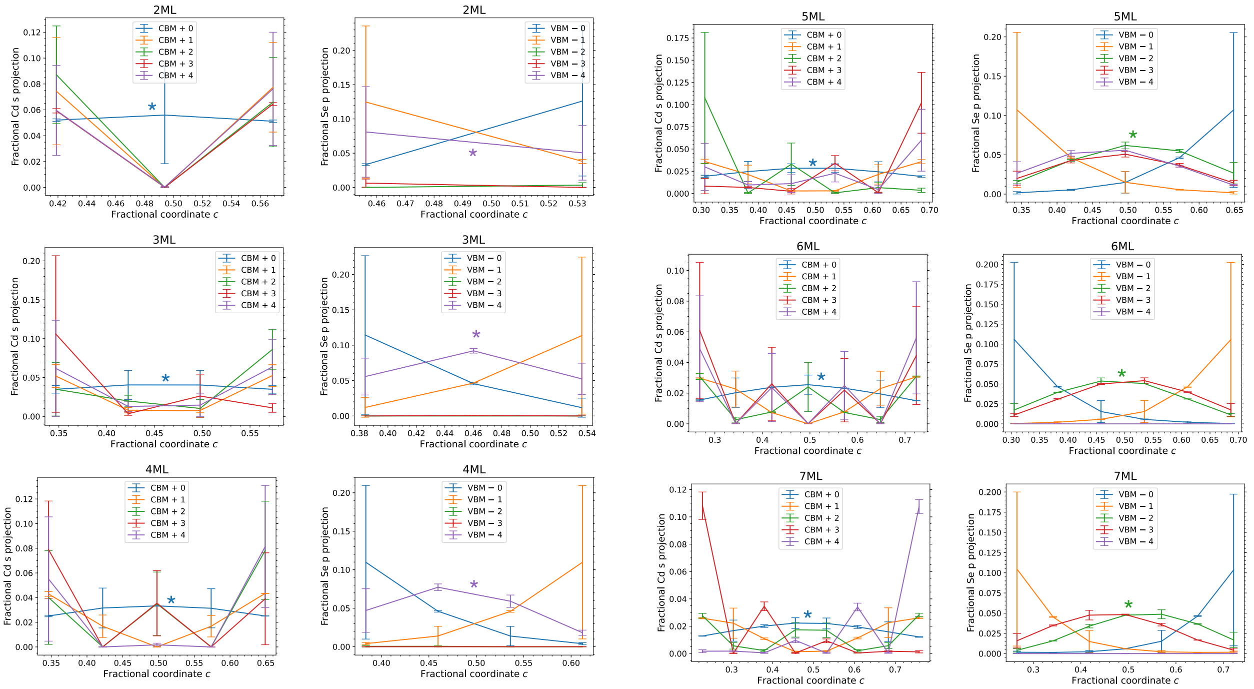

Suppl. Fig. 10. Fractional atomic orbital projections as a function of the relative coordinate along the c direction for S passivation. A color-coded star on each plot labels the band-edge state.

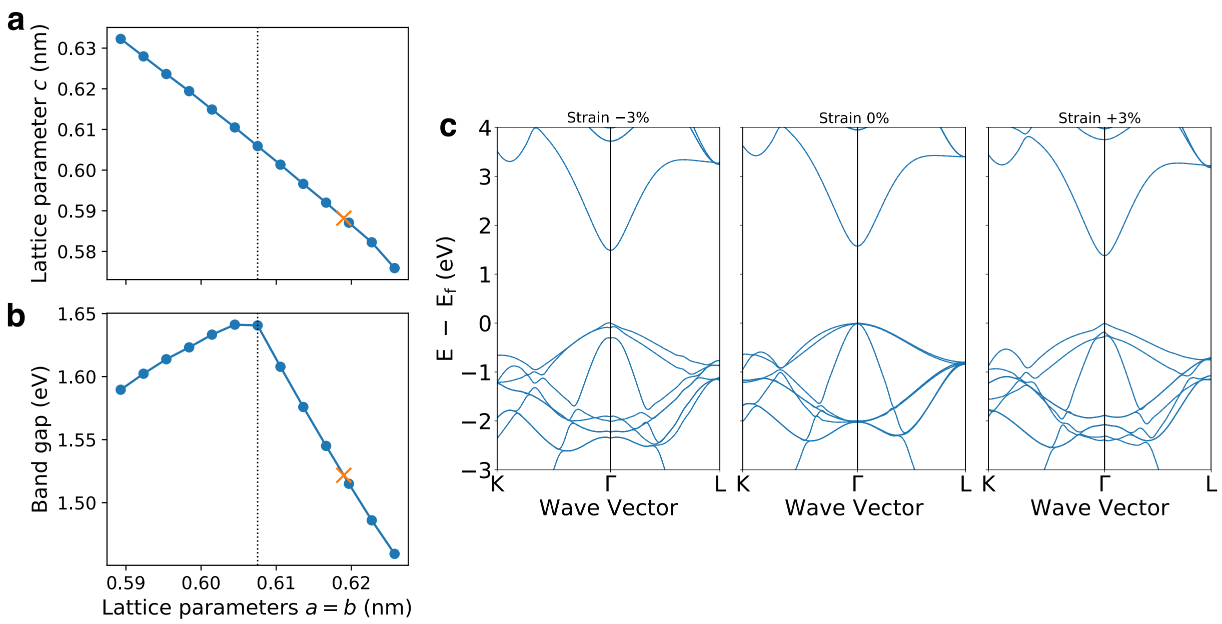

Suppl. Fig. 11. The effect of in-plane strain on CdSe, calculated using HSE. Panel **a** shows the out-of-plane lattice parameter c as a function of the in-plane lattice parameters a and b. Panel **b** shows the resulting band gap. The unstrained parameter is shown as a vertical dashed line. The point corresponding to oleate ligands is shown by an orange x. The gap is maximized at zero strain, and decreases faster for tensile in-plane strain. This may be explained by the effect on the band structure, as shown in panel **c**. In-plane strain splits the threefold-degenerate valence band. Compressive in-plane strain raises two of the bands, while tensile strain raises only one, leading to differing slopes of the band gap versus in-plane strain observed in panel **b**.

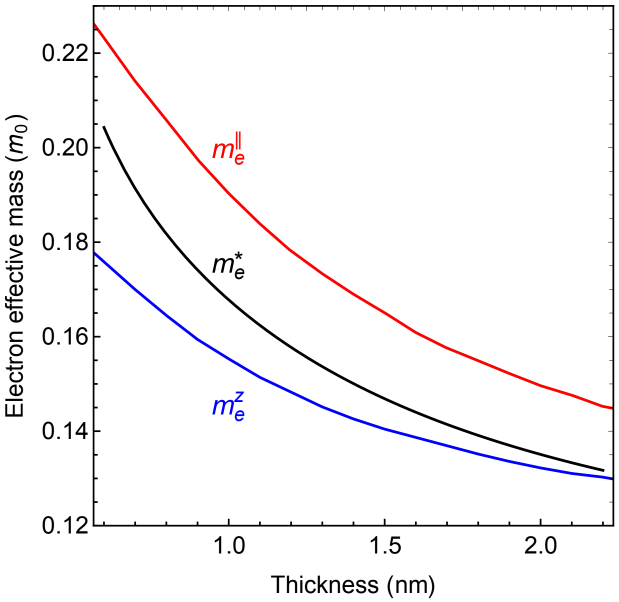

Suppl. Fig. 12. Electron effective masses in-plane ($m_e^{\|}$, red) and out-of-plane (m_e^z, blue), calculated using an 8-band k·p model. [7] Shown in black is m_e^* calculated using k·p via eq. S23, which depends only on the band gap shift in Suppl. Fig. **5a**.

Suppl. Note 4. Thickness dependence of electron effective mass

The effective mass of the hole is energy independent, while the effective mass of the electron depends on the energy due to nonparabolicity of the conduction band [2], increasing with a decrease in the nanoplatelet thickness. The dependence was described in ref. 12 and is reproduced in Suppl. Fig. **1**. We take the hole effective mass to be $m_\mathrm{h} = 0.19 m_0$ [12].

The electron effective masses in-plane ($m_\mathrm{e}^{\|}$) and out-of-plane (m_e^z) were calculated in ref. 7 using an 8-band k·p model. We compare a simple calculation that would predict the mass based purely on the shift in band gap, using equation

$$\frac{m_0}{m_\mathrm{e}^*(d)} = \alpha + \frac{E_\mathrm{p}}{3}\left(\frac{2}{E_\mathrm{g}(d)} + \frac{1}{E_\mathrm{g}(d)+\Delta}\right), \tag{S23}$$

where α is the remote-band contribution, E_p is the Kane energy, $E_\mathrm{g}(d)$ is the thickness-dependent gap (Suppl. Fig. **5a**), and $\Delta = 0.9418$ eV is the spin-orbit split-off energy. To reproduce the bulk electron effective mass of 0.11 m_0 when $E_\mathrm{g} = 1.66$ eV, we set $\alpha = -0.179$. As shown in Suppl. Fig. **12**, this simple model captures the qualitative trend of the full 8-band electron effective mass calculation.

Suppl. Note 5. Average lifetime in ideal nanoplatelets

To calculate the average lifetime in ideal platelets as a function of temperature, we must consider the excited state series for both the dark state (spin forbidden) and the bright state (spin allowed). We use the relation $\tau_{\text{ex}} = \tau_0/\mathcal{D}^2 K$, where K is given by eq. 2 and $\mathcal{D} = 1$ for light at normal incidence because of the platelet geometry. In an ideal square platelet, the K factor for an excited state with quantum numbers N_x and N_y is:

$$K^{\text{B}}(N_x, N_y) = \frac{4L_xL_y}{\pi^4 N_x^2 N_y^2}(1-(-1)^{N_x})(1-(-1)^{N_y}) \tag{S24}$$

Note that the "bright" (spin-allowed) states with even N_x or N_y are momentum forbidden and thus have zero recombination rate. The energy of the state is

$$E^{\text{B}}(N_x, N_y) = \frac{\hbar^2\pi^2}{2ML_xL_y}\frac{1}{N_x^2 + N_y^2} \tag{S25}$$

We assume the dark state rate vanishes ($K^{\text{D}} = 0$). The excited states are separated by the fine-structure splitting:

$$E^{\text{D}}(N_x, N_y) = E^{\text{B}}(N_x, N_y) - \Delta E_{\text{exch}} \ . \tag{S26}$$

The average lifetime is therefore

$$\frac{1}{\tau_{\text{avg}}(T)} = \frac{1}{\tau_0}\left[\sum_{N_x,N_y}\left(K^{\text{B}}(N_x,N_y)e^{-E^{\text{B}}(N_x,N_y)/k_BT} + K^{\text{D}}(N_x,N_y)e^{-E^{\text{D}}(N_x,N_y)/k_BT}\right)\right] \tag{S27}$$

$$\left[\sum_{N_x,N_y}\left(e^{-E^{\text{B}}(N_x,N_y)/k_BT} + e^{-E^{\text{D}}(N_x,N_y)/k_BT}\right)\right]^{-1} \tag{S28}$$

The result is given in Figure **13**. The ideal lifetimes are always shorter than the real lifetimes for a given thickness and temperature due to the effect of ligand fluctuations.

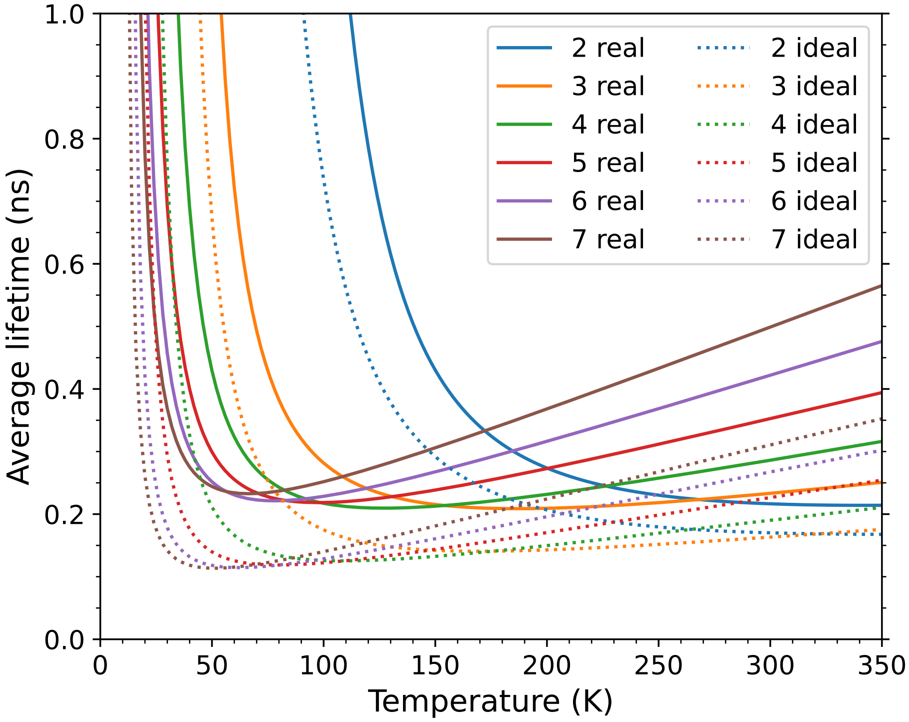

Suppl. Fig. 13. Average lifetime as a function of temperature for CdSe nanoplatelets, color coded for differing thicknesses. Lifetimes for ideal platelets (given by eq. S28) are shown in dashed lines, while lifetimes for real platelets (given by eq. 7) are in solid lines.

Suppl. Note 6. Phonon-induced decoherence

The effects of phonon-induced decoherence on exciton lifetime may be roughly approximated by a phenomenological model, motivated by analogy to a similar model developed for quantum wells by inserting a factor $(1-\gamma_{\rm ph}e^{-\hbar\omega_{\rm ph}/k_BT})$, where $\gamma_{\rm ph}$ is a phenomenological fitting parameter and $\hbar\omega_{\rm ph}$ is the phonon energy, into eq. 7 which describes the average lifetime:

$$\frac{1}{\tau_{\rm avg}(T)} = \frac{(1-\gamma_{\rm ph}e^{-\hbar\omega_{\rm ph}/k_BT})|\phi^d_{\rm 2D}(0)|^2}{\tau_0}\frac{\int A(\varepsilon)\exp(-\varepsilon/kT)d\varepsilon}{\int[n(\varepsilon)+n(\varepsilon+\Delta E_{\rm exch})]\exp(-\varepsilon/kT)d\varepsilon}\ . \qquad \text{(S29)}$$

Assuming $\gamma_{\rm ph}=1$ and using the energy of the laterally confined transverse-acoustic phonon with velocity $v_{\rm t} = 1.54 * 10^5\,{\rm cm/s}$ [54], we obtain the lifetimes in Figure **14**. Room-temperature lifetimes are 1.2, 1.8, 3.0, 4.5, 6.5, and 8.9 nanoseconds for 2 through 7 monolayers respectively, in reasonable agreement with experimental range of 2.5 to 11 ns [19, 20].

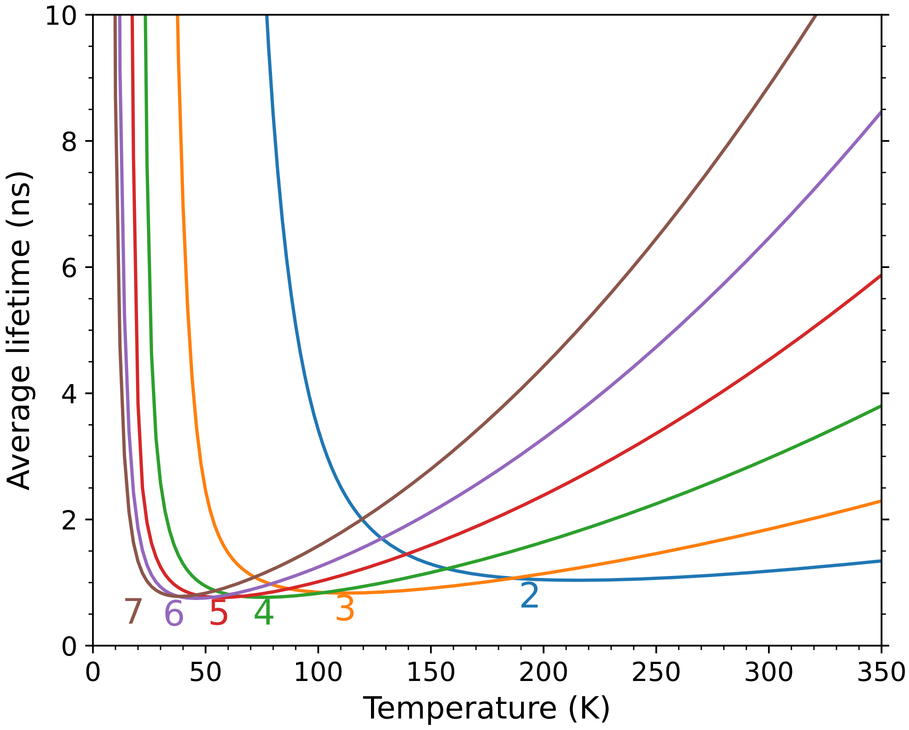

Suppl. Fig. 14. Average lifetime as a function of temperature for CdSe nanoplatelets, including a rough estimate for the effects of phonon-induced decoherence, as described in eq. S29. Lines are color coded for differing thicknesses in monolayers as labeled.

Supplementary References

[1] Ithurria, S. & Dubertret, B. Quasi 2D Colloidal CdSe Platelets with Thicknesses Controlled at the Atomic Level. *J. Am. Chem. Soc.* **130**, 16504–16505 (2008).

[2] Ithurria, S. *et al.* Colloidal nanoplatelets with two-dimensional electronic structure. *Nat. Mater* **10**, 936–941 (2011).

[3] Tyagi, P., Arveson, S. M. & Tisdale, W. A. Colloidal organohalide perovskite nanoplatelets exhibiting quantum confinement. *J. Phys. Chem. Lett.* **6**, 1911–1916 (2015).

[4] Sapori, D., Kepenekian, M., Pedesseau, L., Katan, C. & Even, J. Quantum confinement and dielectric profiles of colloidal nanoplatelets of halide inorganic and hybrid organic–inorganic perovskites. *Nanoscale* **8**, 6369–6378 (2016).

[5] Singh, S. *et al.* Colloidal CdSe Nanoplatelets, A Model for Surface Chemistry/Optoelectronic Property Relations in Semiconductor Nanocrystals. *J. Am. Chem. Soc.* **140**, 13292–13300

(2018).

[6] Cho, W. *et al.* Direct Synthesis of Six-Monolayer (1.9 nm) Thick Zinc-Blende CdSe Nanoplatelets Emitting at 585 nm. *Chem. Mater.* **30**, 6957–6960 (2018).

[7] Shornikova, E. V. *et al.* Addressing the exciton fine structure in colloidal nanocrystals: the case of CdSe nanoplatelets. *Nanoscale* **10**, 646–656 (2018).

[8] Meerbach, C. *et al.* Brightly Luminescent Core/Shell Nanoplatelets with Continuously Tunable Optical Properties. *Advanced Optical Materials* **7**, 1801478 (2019).

[9] Katan, C., Mercier, N. & Even, J. Quantum and Dielectric Confinement Effects in Lower-Dimensional Hybrid Perovskite Semiconductors. *Chem. Rev.* **119**, 3140–3192 (2019).

[10] Shornikova, E. V. *et al.* Negatively Charged Excitons in CdSe Nanoplatelets. *Nano Lett.* **20**, 1370–1377 (2020).

[11] Benchamekh, R. *et al.* Tight-binding calculations of image-charge effects in colloidal nanoscale platelets of CdSe. *Phys. Rev. B* **89**, 035307 (2014).

[12] Shornikova, E. V. *et al.* Exciton Binding Energy in CdSe Nanoplatelets Measured by One- and Two-Photon Absorption. *Nano Lett.* **21**, 10525–10531 (2021).

[13] Efros, Al. L. & Brus, L. E. Nanocrystal Quantum Dots: From Discovery to Modern Development. *ACS Nano* **15**, 6192–6210 (2021).

[14] Gramlich, M. *et al.* Dark and Bright Excitons in Halide Perovskite Nanoplatelets. *Advanced Science* **9**, 2103013 (2022).

[15] Naeem, A. *et al.* Giant exciton oscillator strength and radiatively limited dephasing in two-dimensional platelets. *Phys. Rev. B* **91**, 121302 (2015).

[16] Li, Q., Liu, Q., Schaller, R. D. & Lian, T. Reducing the Optical Gain Threshold in Two-Dimensional CdSe Nanoplatelets by the Giant Oscillator Strength Transition Effect. *J. Phys. Chem. Lett.* **10**, 1624–1632 (2019).

[17] Ji, B. *et al.* Dielectric Confinement and Excitonic Effects in Two-Dimensional Nanoplatelets. *ACS Nano* **14**, 8257–8265 (2020).

[18] Diroll, B. T. & Schaller, R. D. Reexamination of the Giant Oscillator Strength Effect in CdSe Nanoplatelets. *J. Phys. Chem. C* **127**, 4601–4608 (2023).

[19] Di Giacomo, A., Rodà, C., Khan, A. H. & Moreels, I. Colloidal Synthesis of Laterally Confined Blue-Emitting 3.5 Monolayer CdSe Nanoplatelets. *Chem. Mater.* **32**, 9260–9267 (2020).

[20] Christodoulou, S. *et al.* Chloride-Induced Thickness Control in CdSe Nanoplatelets. *Nano Lett.* **18**, 6248–6254 (2018).

[21] Achtstein, A. W. *et al.* Tuning exciton diffusion, mobility and emission line width in CdSe nanoplatelets *via* lateral size. *Nanoscale* **12**, 23521–23531 (2020).

[22] Diroll, B. T., Dabard, C., Lhuillier, E. & Ithurria, S. Band Edge Excitons and Amplified Spontaneous Emission of Mercury Chalcogenide Nanoplatelets. *Advanced Optical Materials* **12**, 2302004 (2024).

[23] Liu, M. *et al.* Colloidal quantum dot electronics. *Nat. Electron.* **4**, 548–558 (2021).

[24] Ahn, N. *et al.* Electrically driven amplified spontaneous emission from colloidal quantum dots. *Nature* **617**, 79–85 (2023).

[25] Tessier, M. D., Javaux, C., Maksimovic, I., Loriette, V. & Dubertret, B. Spectroscopy of Single CdSe Nanoplatelets. *ACS Nano* **6**, 6751–6758 (2012).

[26] Tessier, M. D. *et al.* Phonon Line Emission Revealed by Self-Assembly of Colloidal Nanoplatelets. *ACS Nano* **7**, 3332–3340 (2013).

[27] Achtstein, A. W. *et al.* Electronic Structure and Exciton–Phonon Interaction in Two-Dimensional Colloidal CdSe Nanosheets. *Nano Lett.* **12**, 3151–3157 (2012).

[28] Antolinez, F. V., Rabouw, F. T., Rossinelli, A. A., Cui, J. & Norris, D. J. Observation of Electron Shakeup in CdSe/CdS Core/Shell Nanoplatelets. *Nano Lett.* **19**, 8495–8502 (2019).

[29] Rashba, E. I. & Gurgenishvili, G. E. Edge absorption theory in semiconductors. *Sov. Phys. Solid State* **4**, 759 (1962).

[30] Geiregat, P. *et al.* Localization-limited exciton oscillator strength in colloidal CdSe nanoplatelets revealed by the optically induced stark effect. *Light Sci Appl* **10**, 112 (2021).

[31] Feldmann, J. *et al.* Linewidth dependence of radiative exciton lifetimes in quantum wells. *Phys. Rev. Lett.* **59**, 2337–2340 (1987).

[32] Hanamura, E. & Nagaosa, N. Quantum wells with enhanced exciton effects and optical non-linearity. *Mater. Sci. Eng. B* **1**, 255–258 (1988).

[33] Sercel, P. C. & Efros, Al. L. Band-Edge Exciton in CdSe and Other II–VI and III–V Compound Semiconductor Nanocrystals - Revisited. *Nano Lett.* **18**, 4061–4068 (2018).

[34] Ablyazov, N. N., Raikh, M. E. & Efros, Al. L. Line width of exciton absorption in solid solutions. *Sov. Phys. Solid State* **25**, 199–202 (1983).

[35] Efros, Al. L., Wetzel, C. & Worlock, J. M. Effect of a random adiabatic potential on the optical properties of two-dimensional excitons. *Phys. Rev. B* **52**, 8384–8390 (1995).

[36] Webber, D. H. & Brutchey, R. L. Ligand Exchange on Colloidal CdSe Nanocrystals Using Thermally Labile *tert* -Butylthiol for Improved Photocurrent in Nanocrystal Films. *J. Am. Chem. Soc.* **134**, 1085–1092 (2012).

[37] Diroll, B. T. Ligand-Dependent Tuning of Interband and Intersubband Transitions of Colloidal CdSe Nanoplatelets. *Chem. Mater.* **32**, 5916–5923 (2020).

[38] Antanovich, A. *et al.* A strain-induced exciton transition energy shift in CdSe nanoplatelets: the impact of an organic ligand shell. *Nanoscale* **9**, 18042–18053 (2017).

[39] Riedinger, A. *et al.* An intrinsic growth instability in isotropic materials leads to quasi-two-dimensional nanoplatelets. *Nat. Mater* **16**, 743–748 (2017).

[40] Dufour, M. *et al.* Halide Ligands To Release Strain in Cadmium Chalcogenide Nanoplatelets and Achieve High Brightness. *ACS Nano* **13**, 5326–5334 (2019).

[41] Jana, S., De Frutos, M., Davidson, P. & Abécassis, B. Ligand-induced twisting of nanoplatelets and their self-assembly into chiral ribbons. *Sci. Adv.* **3**, e1701483 (2017).

[42] Vasiliev, R. B. *et al.* Spontaneous Folding of CdTe Nanosheets Induced by Ligand Exchange. *Chem. Mater.* **30**, 1710–1717 (2018).

[43] Monego, D. *et al.* Ligand-induced incompatible curvatures control ultrathin nanoplatelet polymorphism and chirality. *Proc. Natl. Acad. Sci. U.S.A.* **121**, e2316299121 (2024).

[44] Po, H. *et al.* Chiral Helices Formation by Self-Assembled Molecules on Semiconductor Flexible Substrates. *ACS Nano* **16**, 2901–2909 (2022).

[45] Peifer, S., Wiscons, R. A. & Olshansky, J. H. Correlating structural distortions and optical shifts in carboxylate-exchanged CdSe nanoplatelets. *The Journal of Chemical Physics* **158**, 164705 (2023).

[46] Munro, A. M., Jen-La Plante, I., Ng, M. S. & Ginger, D. S. Quantitative Study of the Effects of Surface Ligand Concentration on CdSe Nanocrystal Photoluminescence. *J. Phys. Chem. C* **111**, 6220–6227 (2007).

[47] Boles, M. A., Ling, D., Hyeon, T. & Talapin, D. V. The surface science of nanocrystals. *Nat. Mater* **15**, 364–364 (2016).

[48] Shornikova, E. V. *et al.* Surface spin magnetism controls the polarized exciton emission from CdSe nanoplatelets. *Nat. Nanotechnol.* **15**, 277–282 (2020).

[49] Nirmal, M. *et al.* Observation of the "Dark Exciton" in CdSe Quantum Dots. *Phys. Rev. Lett.* **75**, 3728–3731 (1995).

[50] Efros, Al. L. *et al.* Band-edge exciton in quantum dots of semiconductors with a degenerate valence band: Dark and bright exciton states. *Phys. Rev. B* **54**, 4843–4856 (1996).

[51] Cho, K. Mechanisms for LT splitting of polarization waves: A link between electron-hole exchange interaction and depolarization shift. *J. Phys. Soc. Japan* **68**, 683–691 (1999).

[52] Swift, M. W., Lyons, J. L., Efros, Al. L. & Sercel, P. C. Rashba exciton in a 2D perovskite quantum dot. *Nanoscale* **13**, 16769–16780 (2021).

[53] Biadala, L. *et al.* Recombination Dynamics of Band Edge Excitons in Quasi-Two-Dimensional CdSe Nanoplatelets. *Nano Lett.* **14**, 1134–1139 (2014).

[54] Antolinez, F. V. *et al.* Trion Emission Dominates the Low-Temperature Photoluminescence of CdSe Nanoplatelets. *Nano Lett.* **20**, 5814–5820 (2020).

[55] Diroll, B. T. *et al.* Semiconductor Nanoplatelet Excimers. *Nano Lett.* **18**, 6948–6953 (2018).

[56] Achtstein, A. W. *et al.* p-State Luminescence in CdSe Nanoplatelets: Role of Lateral Confinement and a Longitudinal Optical Phonon Bottleneck. *Phys. Rev. Lett.* **116**, 116802 (2016).

[57] Saviot, L., Champagnon, B., Duval, E., Kudriavtsev, I. & Ekimov, A. Size dependence of acoustic and optical vibrational modes of CdSe nanocrystals in glasses. *J. Non-Cryst. Solids* **197**, 238–246 (1996).

[58] Goupalov, S. V. Low-Frequency Vibrations of Semiconductor Nanoplatelets. *J. Phys. Chem. C* **123**, 11926–11932 (2019).

[59] Ayari, S. *et al.* Tuning trion binding energy and oscillator strength in a laterally finite 2D system: CdSe nanoplatelets as a model system for trion properties. *Nanoscale* **12**, 14448–14458 (2020).

[60] Raikh, M. E. & Efros, Al. L. Broadening of a diamagnetic exciton line of solid solutions. *Sov. Phys. Solid State* **26**, 61–66 (1984).

[61] Thouless, D. J. & Elzain, M. E. The two-dimensional white noise problem and localisation in an inversion layer. *J. Phys. C: Solid State Phys.* **11**, 3425–3438 (1978).
