# Supplementary material for: Controlling light emission from semiconductor nanoplatelets using surface chemistry
Source: Nat Commun. 2024 Sep 4;15:7737. doi: 10.1038/s41467-024-51842-4 (PMC11374790; doi:10.1038/s41467-024-51842-4)
Supplement: Supplementary file 3 — Description of Additional Supplementary Files [file 41467_2024_51842_MOESM3_ESM.pdf]

Description of Additional Supplementary Files:

Supplementary Data 1: Python code implementing the ligand fluctuation model
